# The Role of ERO1α in Modulating Cancer Progression and Immune Escape

**DOI:** 10.33696/cancerimmunol.2.023

**Published:** 2020

**Authors:** Brennan D. Johnson, Werner J. Geldenhuys, Lori A. Hazlehurst

**Affiliations:** 1 WVU Cancer Institute, Morgantown, WV 26506, USA; 2 WVU School of Pharmacy, Morgantown, WV, 25606, USA; 3 WVU Neuroscience Institute, Morgantown, WV, 25606, USA

**Keywords:** ERO1, Cancer, ER stress, Cancer therapeutics, Immune resistance

## Abstract

Endoplasmic reticulum oxidoreductin-1 alpha (ERO1α) was originally shown to be an endoplasmic reticulum (ER) resident protein undergoing oxidative cycles in concert with protein disulfide isomerase (PDI) to promote proper protein folding and to maintain homeostasis within the ER. ERO1α belongs to the flavoprotein family containing a flavin adenine dinucleotide utilized in transferring of electrons during oxidation-reduction cycles. This family is used to maintain redox potentials and protein homeostasis within the ER. ERO1α’s location and function has since been shown to exist beyond the ER. Originally thought to exist solely in the ER, it has since been found to exist in the golgi apparatus, as well as in exosomes purified from patient samples. Besides aiding in protein folding of transmembrane and secretory proteins in conjunction with PDI, ERO1α is also known for formation of *de novo* disulfide bridges. Public databases, such as the Cancer Genome Atlas (TCGA) and The Protein Atlas, reveal ERO1α as a poor prognostic marker in multiple disease settings. Recent evidence indicates that ERO1α expression in tumor cells is a critical determinant of metastasis. However, the impact of increased ERO1α expression in tumor cells extends into the tumor microenvironment. Secretory proteins requiring ERO1α expression for proper folding have been implicated as being involved in immune escape through promotion of upregulation of programmed death ligand-1 (PD-L1) and stimulation of polymorphonuclear myeloid derived suppressor cells (PMN-MDSC’s) via secretion of granulocytic colony stimulating factor (G-CSF). Hereby, ERO1α plays a pivotal role in cancer progression and potentially immune escape; making ERO1α an emerging attractive putative target for the treatment of cancer.

## Introduction

**Figure 1:**
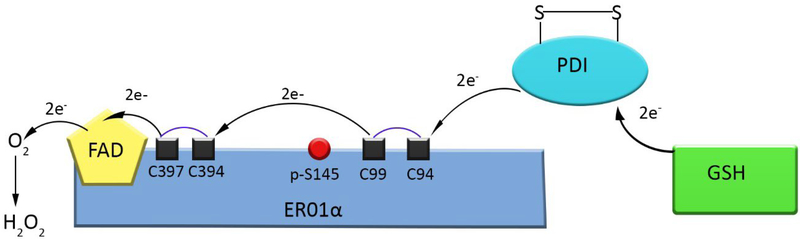
Upon Phosphorylation of Serine 145 by FAM20C Kinase, ERO1α can return to the endoplasmic reticulum (ER). Above is an illustration of the interaction occurring between active site of ERO1α, PDI, and Glutathione (GSH). This reaction is also capable of occurring in the reverse direction resulting in redox equilibrium inside the ER. (Red circle is the phosphorylation site of ERO1α at serine 145 via FAM20C kinase, Black squares connected by blue lines are active disulfide bridges required for redox to occur, and black arrows are representative of electron flow when ERO1α is being reduced.

The failure to eradicate minimal residual disease often located at metastatic sites and/or the bone marrow niche continues to be a clinical barrier for successful treatments in cancer [1–4]. Unfortunately, in some tumor’s disease relapse is associated with a multi-drug resistant phenotype that corresponds to resistance to structurally and functionally divergent agents. Similar to conventional therapies the success of immune-oncology is limited to the occurrence of primary resistance in some patients, as well as emergence of acquired resistance [5]. Accumulating experimental evidence indicates that the tumor microenvironment plays a critical role in mediating sensitivity to targeted agents as well as immunotherapy [6,7]. Thus, to improve the gap in patient outcomes new targets need to be validated in the context of the metastatic phenotype and the tumor microenvironment. This review will discuss the potential of ERO1α as target for the treatment of cancer. Despite the endoplasmic reticulum being one of the largest cellular organelles, it was one of the last ones discovered [8]. Originally described by Emilio Veratti in 1902, it was not until the electron microscope was available that George Palade and Keith Porter made the rediscovery [9–11] capturing the structural complexities and tubular structure existing in the cytoplasm [12]. Since the re-discovery, the ER has been identified to be a continuous membranous organelle essential for protein folding, calcium storage, lipid metabolism, protein transport, post-translational modifications, and protein transport via vesicles [13]. It is composed of two main parts; smooth ER and rough ER. The rough ER is composed of ribosomes and continuous cisternae that have an important role in protein folding and storage, while the smooth ER is void of ribosomes and composed mainly of microtubules, and is critical for synthesis and storage of lipids. Maintaining homeostasis within the ER is essential for proper formation of desulphated bridges and ultimately, protein folding [14]. A major determinant of homeostasis occurs through oxidative enzymes of the flavin dependent endoplasmic reticulum oxidoreductin-1 (ERO1α) family [15–19] and by the buffering capacity of reduced glutathione (GSH) and oxidized glutathione (GSSG) in a 3:1–6:1 molar ratio in favor of reduced glutathione [20–22]. ERO1α is known to oxidize protein disulfide isomerase (PDI) in order to form *de novo* disulfide bridges [16]. The crucial function of disulfide bridging occurring in the ER between ERO1α and PDI have been well-established [23]. Briefly, the target unfolded protein within the ER can be oxidized by PDI resulting in the reduction of PDI. Oxidized ERO1α can in turn lead to the recycling of PDI to the oxidized from. Reduced ERO1α can be oxidized and form de-novo disulfide bridge utilizing FAD as a cofactor leading to FADH and reduction of molecular oxygen to ROS (See Figure 1).

Cancer cells typically are under increased levels of ER stress. ER stress is most evident in secretory tumors such as multiple myeloma, breast, lung and pancreatic. However, other inducers of ER stress include hypoxia and chemotherapy. Given the importance of ER homeostasis, ERO1α-PDI interaction, and formation of *de novo* disulfide bridges ERO1α has emerged as a player in the regulation and tolerance of cancer cells to ER stress [24]. An increase in ER-stress can lead to two possible outcomes: 1.) unfolded protein response (UPR) once activated ultimately brings ER stress back to homeostasis via inhibition of translation and inducing cell cycle arrest, or 2.) if ER stress is not resolved than apoptosis occurs through release of cytochrome C and caspase activation, ultimately protecting the organism from rogue cells that display misfolded proteins [25]. Despite UPR being the main pathway to ER stress resolution; UPR has also been linked to autophagic flux. Autophagy can either be inhibited or activated upon ER stress [26]. Though autophagy is related to ER stress it is not always activated under ER stress conditions. Autophagy is an orchestrated process by which misfolded proteins, damaged or aged organelles, or even mutated proteins are sequestered in an autophagosome that ultimately fuses to the lysosome leading to degradation of sequestered components [27]. Reports recently have showed that withanolide E in combination with ER stress inducers enhance apoptosis synergistically in pancreatic cancer models [28]. Multiple cancer types have been reported to have increased ER stress including multiple myeloma, lung, breast, and pancreatic [29–31]. Differences in ER stress can be driven by genetic, epigenetic, and microenvironmental heterogeneity that likely result in a range of pro-survival and anti-apoptotic responses [32]. Anticancer interventions such as chemotherapy has also been shown to modulate UPR (Unfolded protein response) though clinical implications are only starting to be understood [33,34]. Recent studies have showed that depending on the context cancer cells can utilize UPR as a resistance mechanism [35].

Interestingly, overexpression of ERO1α tends to have a worse prognosis in multiple cancer indications; multiple myeloma [36], breast [37–39] and hepatocellular carcinoma [40], as well as lung, esophageal, diffuse B-cell lymphoma, and others according to The Cancer Genome Atlas (TCGA) and The Protein Atlas. These data indicate that expression is increased in aggressive and/or drug resistant disease and support the premise the ERO1α is a tractable target for the treatment of cancer. Below, we will delve into four specific topics that will reveal and shed light onto 1.) the structure, function, localization, and characterization of flavin containing ERO1α, 2.) Disease indications, 3.) immune surveillance, and immune evasion and how they are related to ERO1α and 4) chemical probes currently available to test the ERO1α-PDI pathway.

## Structure, Function, Localization, and Characterization of Flavin Containing ERO1α

**Figure 2:**
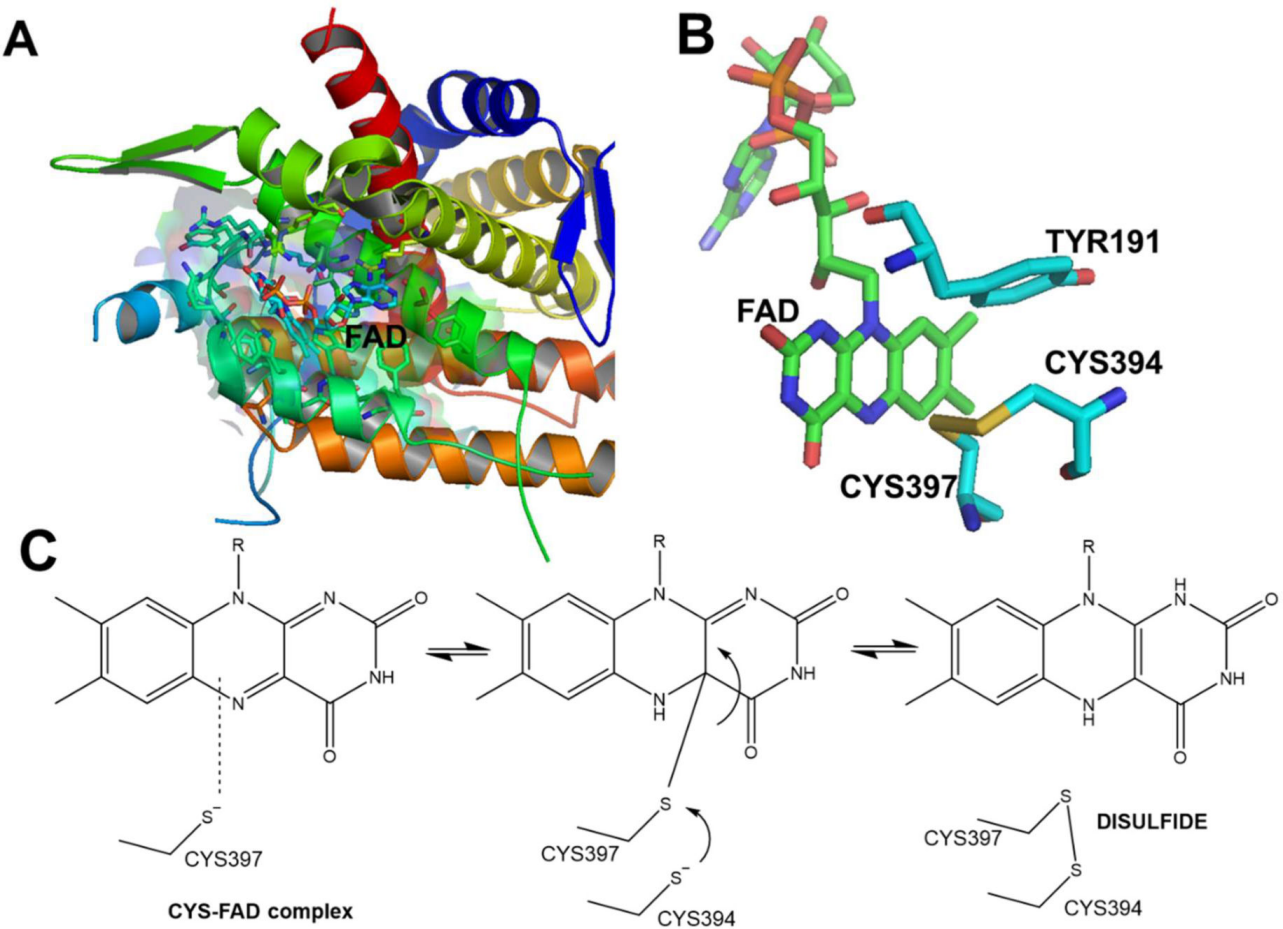
**A)** The structure of ERO1a (3AHQ.pdb) with the FAD binding site shown; **B)** the orientation of the Cys394-Cys397 with FAD; **C)** the proposed catalytic scheme of FAD and disulfide bond formation.

Flavoenzymes are an important classification of enzymes that utilize FAD in redox reactions to maintain enzymatic function. Specifically, ERO1α promotes oxidative protein folding through PDI, producing hydrogen peroxide as a by-product and is tightly regulated to avoid futile oxidation cycles occurring in the ER [41]. There are many secretory and membrane proteins under the assistance of thiol-disulfide oxidoreductases helping form disulfide bonds in the ER [42–46]. The major players in ER oxidative reactions are ERO1α and PDI; both which are conserved from yeast to mammals [16,17,47,48]. Humans contain two ERO1 isoforms; ERO1α that is expressed in almost all cell types and ERO1β that is only expressed in select tissues. The oxidative reaction occurring between ERO1α and PDI produces hydrogen peroxide, a reactive oxygen species (ROS) [43]. Although a cell can cope with peroxides formed during basal oxidative protein folding, sometimes using them as secondary messengers in cell-signaling cascades [49] and possibly as a direct protein disulfide introducer [50,51]. If ROS production exceeds cellular capacity of antioxidants defense systems this can be harmful via introduction of ER oxidative stress [41]. ERO1α is tightly regulated not only through phosphorylation state [52], but also through regulatory disulfide bridging. When disulfide bridges are formed between cysteine 94 and cysteine 99 ERO1α activity exceeds WT ERO1α (known as hyperactive ERO1α), whereas if cysteine 94 is bridged with cysteine 131 it leads to complete inactivity of ERO1α (Inactive ERO1α) [41]. This was shown using biochemical approaches through site-directed mutagenesis in yeast. However, this provides a potential regulatory or even compensatory mechanism that ERO1α can exploit when the ER is under extreme stress conditions. Hyperactive, inactive, and WT are the three forms of ERO1α that have been shown to exist in yeast. In yeast, cysteine 100, cysteine 105, cysteine 352, and cysteine 355 are required for oxidative reactions, whereas cysteines 90, 208, and 349 are dispensable for these functions [17]. Providing a mechanism by which cysteine 100-cysteine 105 directly engage in oxidative reactions; whereas cysteine 352-cysteine 355 serve directly to re-oxidize cysteine 100-cysteine 105. Allowing for ERO1p to undergo another oxidative reaction [16,17,41]. In humans, ERO1α catalyzes the formation of a cysteine disulfide bond as part of the FAD containing enzyme. As can be seen in Figure 2, the cysteine 397 forms a transition complex with FAD, after which cysteine 94 attacks via a nucleophilic C-S bond to form the disulfide bond, with FAD reduced to the FADH_2._ Cysteine 352-Cysteine 355 bridge in yeast are equivalent to Cysteine 394-Cysteine 397 in humans and undergo the same reaction process with the cofactor FAD. The regulatory cysteine bridges in yeasts (Cys100-Cys105), is also conserved in humans, and is the disulfide bridge occurring between cysteine 94-cysteine 99. This disulfide bridge in humans are responsible for accepting electrons from PDI and transferring them to the disulfide bridge between cysteine 394-cysteine 397; ultimately allowing for cysteine 397 to perform the nucleophilic attack onto bound FAD (see Figure 1 and Figure 2).

Upon successful oxidative protein folding, basal ERO1α can be shuttled into the golgi apparatus, where an interaction with FAM20C kinase occurs [52]. FAM20C is a secretory kinase founded in 2012 known for phosphorylation of the secretory protein Casein. Interestingly FAM20C prefers to use Manganese (Mn^2+^) instead of Magnesium (Mg^2+^) as compared to other kinases and is known for recognizing S-x-E/pS motif of secretory proteins [53,54]. It has also been shown to be insensitive to staurosporine, a known broad-spectrum kinase inhibitor. Recently, FAM20C was shown to phosphorylate ERO1α at serine residue 145 (S145). This phosphorylation state allows for 1 of 2 scenarios to take place; 1.) ERO1α can be sent extracellularly through packaging into exosomes, or 2.) Is sequestered by ERp44 (an ER transporter and chaperone protein primarily located in the endoplasmic reticulum-golgi intermediate compartment (ER-GIC) to be transferred back into the ER to undergo another oxidation cycle with PDI [55]. Zhang et al. was also able to conclude from their study that ERO1α activity was increased upon phosphorylation of residue S145, and that this reaction takes place during mammalian lactation, under hypoxia, and reductive stress conditions. Originally reported to co-localize with PDI in the ER lumen [16], ERO1α has more recently been shown to localize in the golgi apparatus [52], in proximity to the mitochondrial associated Endoplasmic Reticulum membranes (MAM), but only under oxidizing and normoxic conditions [56], and was identified from purified exosomes from bladder cancer cells, liver cancer cells, and squamous cell carcinoma cells (exocarto and protein atlas). More intriguing, ERO1α under basal conditions is still found to be localized in the ER despite not having a peptide signal sequence such as the C-terminus KDEL like other ER-resident proteins [57]. The absence of an ER localization signal suggests that ERO1α functions may extend beyond the ER and these additional functions based on localization of the enzyme need to be discovered in order to fully understand role of ERO1α in the progression of cancer. Questions still needing to be answered are 1.) What function does ERO1α provide by being packaged into exosomes or by remaining in the golgi apparatus, and 2.) What function does ERO1α have when localized to the MAM region? Though these questions have not been answered yet fully, there has been some insight onto the function of ERO1α when localized near the MAM region. This function is to regulate Ca^2+^ flux. Downregulation of ERO1α via RNAi was found to inhibit mitochondrial Ca^2+^ fluxes and modified mitochondrial Ca^2+^ uniporters. However, upon overexpression of redox active ERO1α increased passive Ca^2+^ efflux from the ER was observed [58]. Calcium flux is an essential ER regulated process that is used in signaling, activation of apoptosis, and even used in cellular movement. Calcium is stored in the ER but released into the mitochondria for activation of apoptosis. Calcium can be transferred from the ER into the mitochondria via the MAM region and is required to maintain cellular homeostasis [59]. Thus, ERO1α functionality beyond the scope of just protein folding in the ER as localization can play pivotal roles in regulating protein function.

## Disease Indications

Recently, ERO1α has been reported as a poor prognostic indicator in multiple cancer indications. Yang et al, showed using genetic shRNA strategies for reducing the expression of Ero1α, in HepG2 and Hep3B cells that high ERO1α expression correlated with increased migration and invasion. Moreover, these same investigators showed that in primary patient specimens high ERO1α expression was associated with poor clinicopathology of vascular invasion, metastasis, advanced Edmondson grade, and TNM stage [40]. Yang et al. were also able to conclude from their *in vivo* studies using HepG2 cells ectopically expressing ERO1α that an increase in metastatic burden and poor survival *in vivo* correlated with increased ERO1α expression and S1PR1, p-STAT3, and VEGF-A levels. However, upon depletion of ERO1α using shRNA strategies, S1PR1, p-STAT3, and VEGF-A were also reduced.

**Figure 3:**
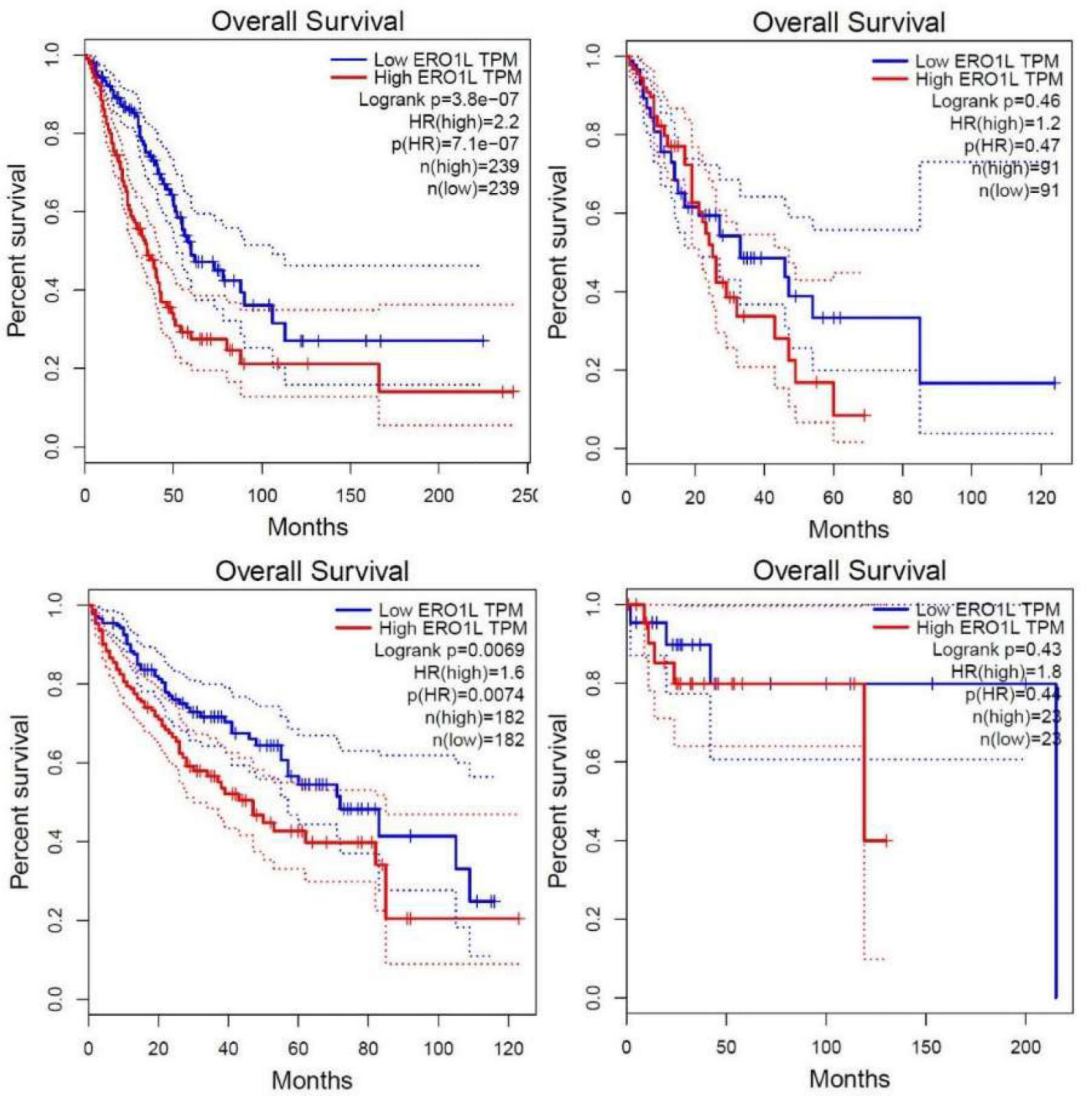
Top Left Panel represents survival data for Lung Adenocarcinoma in respect to ERO1α, Top Right Panel represents Esophageal Carcinoma, Bottom Left Panel represents survival data for hepatocellular carcinoma, and Bottom Right Panel represents survival data for diffuse B-cell lymphoma. Kaplan-Meyer Plots above were plotted using ERO1α gene filter in GEPIA, which obtains data from TCGA and GTEx databases.

In support of clinical data indicating that ERO1α expression is a poor prognostic indicator, we probed the GEPIA database that utilizes samples from TCGA and GTEX database and compiles the data into a Kaplan-Meyer plot based on a single gene of interest. Using GEPIA, we determined that that ERO1α is considered a bad prognostic indicator in multiple cancer indications including Lung Adenocarcinoma, Hepatocellular Carcinoma, Esophageal Carcinoma, and Diffuse B-Cell Lymphoma (see Figure 3 and Table 1). Moreover, genetic and pharmacological studies in cell lines has indicated that ERO1α expression and function contributes to an aggressive cancer phenotype (see Table 1).

The GEPIA was able to confirm that high ERO1α transcript levels correlate with worse prognosis in multiple cancer indications. As mentioned previously ERO1α is responsible for protein folding. More specifically, ERO1α is directly involved in folding of membrane and secretory proteins [64]. Recently, ERO1α has also been shown to have a role in post-translational modification of β1 integrin in colorectal cancer cell when under hypoxic conditions. When ERO1α is knocked out using CRISPR Cas9 in HCT116 colorectal cancer cells and placed under hypoxic conditions it was found that the glycosylation state of integrin β1 was changed and thus an attenuation of integrin β1 on the cell membrane occurred; ultimately leading to contact-inhibited morphology [57]. These are just a few examples of many that ERO1α is associated with cancer indications.

## Immune Surveillance and Immune Evasion Correlate with ERO1α Expression

Recently, immuno-oncology (IO) has demonstrated to be a tractable strategy for achieving durable responses. Targets and delivery approaches which may enhance IO response with respect to percentage of patients that respond remains an active area of research (rev in [65]). As IO approaches move toward combination strategies, it is essential to determine the effect of modulating novel targets such as Ero1α on the immune tumor microenvironment. Immunotherapies have previously failed in lung cancer but has recently emerged as very effective new therapy [66], with the emergence of immune checkpoint blockade such as anti-PD-1 (programmed cell death-1) antibodies and anti PD-L1 (programmed cell death-ligand 1) antibodies [67]. ERO1α is thought to be responsible for the processing and folding of PD-L1 and PD-1. PD-L1 is a transmembrane protein located on the cell surface of placental, vascular endothelium, pancreatic islet, muscle, and mesenchymal stem cells [68], and PD-1 is a receptor belonging to the CD28 family of receptors that is only expressed on the surface of activated T-cells, B-cells, and myeloid cells [69]. The binding of PD-L1 to PD-1 is an immune suppressive signal that inhibits autoimmunity through induction of T-cell apoptosis [70] and induces tolerance [71]. Recently, a study has shown that ERO1α promotes immune escape through up-regulation of PD-L1 in breast cancer [60]. This connection is due to the intramolecular disulfide bond that exist in PD-L1 [72] and ERO1α is a known contributor to folding of membrane proteins as well as introduction of disulfide bonds [16]. In this recent finding, Tanaka et al. [60], was able to demonstrate that ERO1α up-regulates PD-L1 surface expression not only through oxidative folding, but also unexpectedly up-regulated PD-L1 mRNA expression through augmentation of hypoxia inducible factor-1alpha (HIF-1α) in human triple negative breast cancer cell lines. Although this occurrence could be diminished upon knockdown of ERO1α using RNA Interference (RNAi), as it led to significant attenuation of PD-L1 mediated T-cell apoptosis [60]. This provides insight towards hypoxia mediated immune resistance; specifically, in triple negative breast cancer cell lines. As shown previously in Figure 2, increased expression of ERO1α is a poor prognostic indicator in lung adenocarcinoma, hepatocellular carcinoma, esophageal carcinoma, and diffuse B-cell lymphoma; However, ERO1α has also be found to be a poor prognostic indicator in breast cancer by multiple researchers [37,38].

Alongside the discovery of PD-L1 and PD-1, of which Dr. Allison and Dr. Honjo won the 2018 Nobel Prize for the discovery of checkpoint blockades PD-1, PD-L1, and CTLA-4, the discovery of myeloid derived suppressor cells (MDSC’s) has also had an outstanding impact clinically. Reports of MDSC’s associated with tumor progression go back to the 1970’s [73]. However, during the 1980’s and early 1990’s, laboratories of Diana Lopez, Jim Talmadge, M. Rita Young, and Hans Schreiber, demonstrated various types of myeloid cells could inhibit immune functions in cancer [74]. There are two main groups of MDSC’s; Polymorphonuclear MDSC’s (PMN-MDSC’s), and monocytic MDSC’s (M-MDSC’s). In recent years it has become clear that these two groups function differently in terms of immune suppression during tumorigenesis. M-MDSC’s suppress the immune system in both antigen-specific and non-specific manners utilizing mechanisms associated with production of NO and cytokines [75]. PMN-MDSC’s on the other hand can suppress immune responses primarily in an antigen-specific manner, inducing antigen specific T-cell tolerance is a major characteristic of these cells [76,77]. Recently it has been shown that tumor cells are a source of granulocytic colony stimulating factor (G-CSF) [78–81], and that production of G-CSF by tumors are responsible for recruitment of immunosuppressive PMN-MDSC’s, which promote tumor growth via inhibition of antitumor immune responses [82,83]. G-CSF is a glycoprotein that functions as a hematopoietic cytokine that is secreted from immune, endothelial and bone marrow stroma cells leading to production of granulocytes (granulopoiesis), as well as contributes to mobilization of stem cells [84]. Amongst G-CSF’s many functions, it is a secretory protein that’s folding cycle is predicted to occur through ERO1α. In a recent publication; Tanaka et al. [37] demonstrated that ERO1α plays a pivotal role in PMN-MDSC induction via up-regulation of G-CSF production from cancer cells in collaboration with PDI. Tanaka et al. [37] were also able to demonstrate that reduced expression of ERO1α through shRNA strategies reduced tumor growth by restoration of antitumor T-cell-mediated immunity, and ERO1α overexpression promoted tumor growth *in vivo* via suppression of antitumor immunity.

Immune system functionality has been well defined for quite some time. Despite immune system functionality being well described, cancer researchers are still discovering resistance mechanisms to cancer therapies that are utilizing the host immune system. Recently, it was found that hypoxia augmented the endogenous major histocompatibility complex I (MHC Class I) presentation in murine tumor cells [85]. MHC Class I molecules are responsible for presentation of endogenous antigens, expressed on all nucleated cells, and present protein fragment of cytosolic or nucleic nature to CD8^+^ T-cells on the cell membrane. [86]. These antigens are peptide fragments that are intracellular and obtained from multiple pathways being approximately 8–10 amino acids long [87]. MHC Class I molecules are stabilized by ER chaperones such as ERp57, PDI, and tapasin [86]. Upon binding of the designated peptide antigen to the MHC Class I molecule, the chaperones are released and fully assembled, peptide-MHC Class I complexes leave the ER for presentation of the cell membrane [87]. Conversely, the MHC-Class I peptide complexes that fail to associate in the ER are sent to the cytosol to undergo proteasomal mediated degradation [88,89]. Though the function of MHC Class 1 is clear, it is not clear if this function is augmented in tumors or during hypoxic conditions. HIF-1α is known to be induced under hypoxic conditions and more recently has been shown to regulate expression of ERO1α [90,91]. More specifically, ERO1α was induced in hypoxic conditions in a HIF-1α dependent manner [91]. May et al. [91] were also able to demonstrate that ERO1α was not induced under hypoxic conditions in fibroblast cell lines derived from HIF-1α knockout mice, revealing ERO1α as a transcriptional target of HIF-1α. To induce an effective antitumor immunity using cancer antigen peptide-based immunotherapy, a cancer antigen must be appropriately presented on MHC Class I molecules [37]. Kukita et al. [37] were able to determine three distinct effects that hypoxia had on MHC Class 1 presentation; 1.) expression of MHC Class I peptide complex on the cell surface was augmented, 2.) activation of antigen specific CD8^+^ T-cells was augmented, and 3.) specific cytotoxic T-lymphocytes were capable of killing tumor cells under hypoxic conditions. They were also able to determine that ERO1α was responsible for the hypoxia driven antigen presentation by MHC Class I molecules. ERO1α is responsible for the disulfide bond formation of MHC Class I heavy chains. Upon depletion of ERO1α, MHC Class I expression on the cell surface was also shown to be decreased, resulting in decreased cytotoxic T-lymphocyte reactivity [37]. Thus, Kukita et al [37] were able to demonstrate that ERO1α plays a crucial role in hypoxia-induced oxidative folding of MHC Class I heavy chain, leading to augmentation of MHC Class I-peptide complex on the tumor cell surface and enhanced recognition by antigen specific cytotoxic T-lymphocytes. Functional MHC Class I expression is needed to induce cell death via induction of CD8^+^, T-lymphocytes. Despite increased MHC Class I expression in hypoxic conditions, the increased and chronic presence of cancer associated antigens may lead to T-cell exhaustion in hypoxic regions of the tumor, albeit further work is required to fully determine whether increased Ero1α expression leads to T-cell exhaustion *in vivo* [92, 93]. Because inhibiting Ero1α is likely to change the cytokine and chemokine profile of the tumor microenvironment, further studies are required to fully understand the effect of inhibiting Ero1α on tumor mediated immune suppression.

## Inhibitors of the ERO1 Pathway

**Figure 4:**
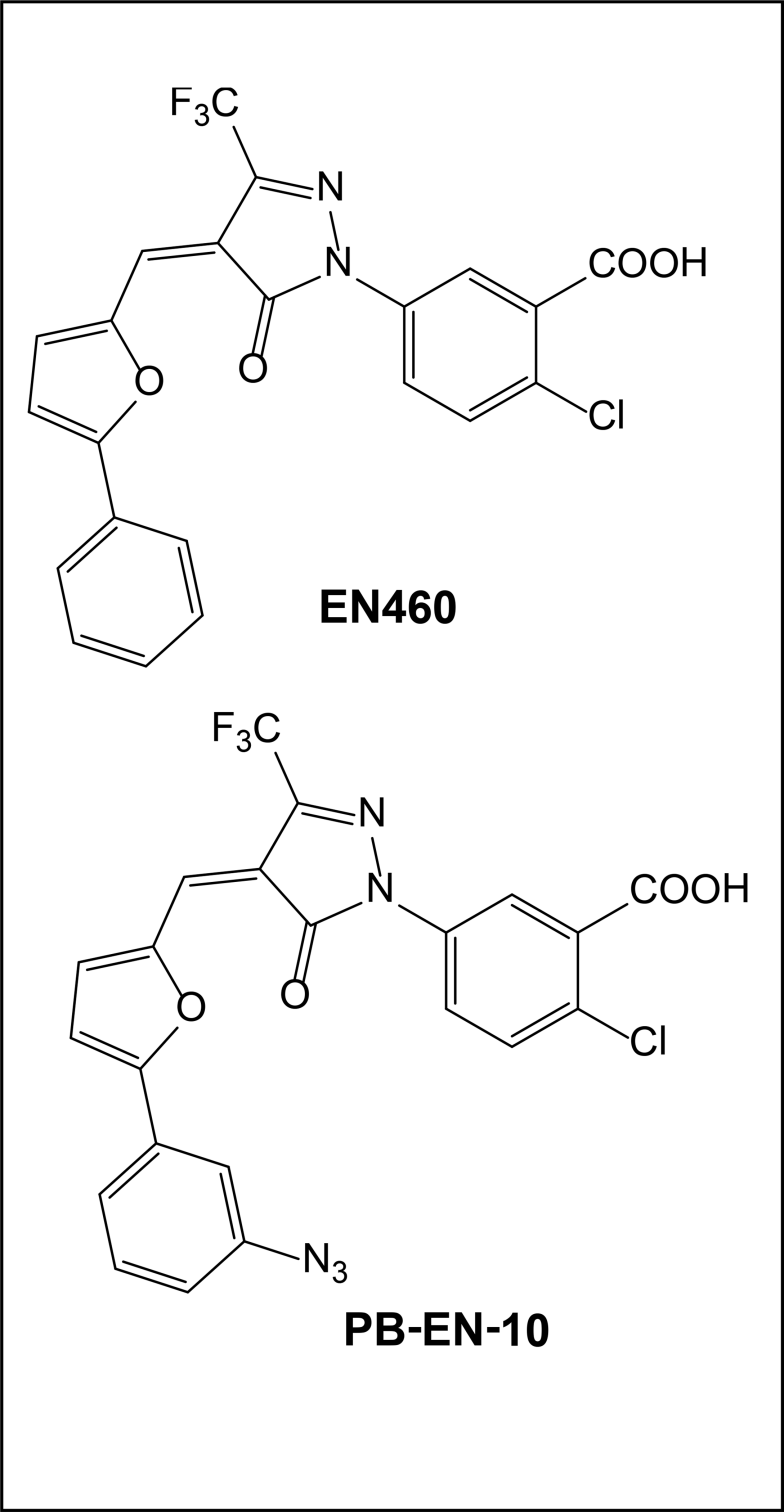
Structure of EN460, the first ERO1α inhibitor, and the azide derivative PB-EN-10.

Currently pharmacologic inhibitors that target ERO1α are limited. The first inhibitor of ERO1α, known as EN460, and was identified through a screen of 210,960 natural compounds [94]. EN460 is specific for the reduced active form of ERO1α and prevents re-oxidation [94]. Our laboratory recently demonstrated that EN460 could potentially be used to treat cancers with high ER-stress such as multiple myeloma [36]. It was also confirmed during this study that EN460 had multiple off targets; all being flavoenzymes, or enzymes that contain flavin adenine dinucleotide (FAD). Our laboratory was also able to develop an azide derivative of EN-460, PB-EN-10, that showed similar effects [36]. The tool compound EN460 and its azide derivative PB-EN-10 are shown below in Figure 4. Having direct interactions with PDI during oxidative protein folding it seems feasible that PDI could also be targeted to inhibited ERO1α mediated biological functions. PDI has also been shown to be a potential target in multiple myeloma. Targeting PDI provides its’ own challenges as it has multiple isoforms being a family with greater than 20 members, and due to the multiple redox active cysteine residues present [95–98]. PDI inhibition currently via small molecules occurs through covalent catalysis and includes the following tool set 16F16, PACMA31, KSC-34, E61, and E64FC26 [99–102]. Despite multiple known inhibitors against PDI, none have entered clinical trials for the treatment of cancer at this time. ERO1α and PDI could be good targets in cancer indications if specificity could be achieved toward both enzymes, as PDI inhibitors tend to hit all PDI family members and ERO1α inhibitors have the tendency to target other flavoenzymes. In conclusion ERO1α is emerging as an attractive target for the treatment of cancer. Supporting evidence that credentials the target described above includes i) clinical data indicating that increased expression of the enzyme is a poor prognostic indicator in multiple cancer indications and ii) genetic strategies utilizing shRNA indicate that reducing the expression of ERO1Lα inhibits growth and metastasis using both *in vitro* and *in vivo* model systems. However, delineation of the therapeutic window will require a drug discovery campaign to elucidate more specific and potent inhibitors to allow for further validation of the target.

## Figures and Tables

**Table 1:** Role of ERO1α in cancer progression and metastatic potential in cancer.

Cancer Type	Cell Type	Native ERO1α expression	Tools used	Native expression effect/silencing effect	Reference’(s)
*Breast*	9A3, 9C1, 9C2,	Comparable to MDA-MB-231	↑ERO1α ↓ERO1α/shRNA	↑ PD-L1; dependent on oxidative folding	[60]
	MDA-MB-231, 9A3, 9C1, 9C2	Comparable to MDA-MB-231	↑ERO1α ↓ ERO1α/shRNA	↑ angiogenic potential via VEGF secretion, ↑ tumor growth	[39]
	MCF10A	Comparable to MCF-7	-	Tumor associated Macrophages increased ERO1α expression, causing ↑MMP-9 expression, and ↑ invasion	[61]
	4T1	Comparable to healthy mouse mammary tissue	↑ ERO1α ↓ ERO1α/shRNA	↓ ERO1α expression lead to ↓ tumor burden: ↑ ERO1α ↑ MDSC’s and leads to immune evasion	[37]
	4T1 Triple negative patient samples	Comparable to healthy mouse mammary tissue; Healthy human breast tissue	↓ ERO1α/shRNA	↓ tumor burden, ↓ lung metastasis; Patients with ↑ ERO1α expression had worse prognosis overall.	[38]
	MDA-MB-231	Comparable to MDA-MB-468	-	MTH-3 treatment lead to ↓ ERO1α, activation of autophagy, and lead to apoptosis	[62]
*Hepatocellular Carcinoma*	Patient samples, LO2, Huh-7, HEP3B, SMMC-7721, HEPG2, MHCC-97H	ERO1α expression lower in healthy tissue compared to tumor samples and cell lines	↓ ERO1α/shRNA ↑ ERO1α	↓ ERO1α lead to less metastasis and decreased tumor burden. ↑ ERO1α lead to increase metastatic potential and increased tumor burden.	[40]
*Head and Neck*	HN4, CAL27	-	-	Tunicamycin treatment ↑ER stress, ↑ ERO1α expression, and ↓overall tumor burden	[63]
*Multiple Myeloma*	Patient samples U266, MM1.S	Increased ERO1α expression poor prognostic indicator in multiple myeloma	Chemical EN-460 PB-EN-10	Pharmacological inhibition Ero1α lead apoptosis and ER stress in U266 myeloma cell line	[36]

(-) indicates no available data
